# Supplementary material for: Evaluation of a mobile application tool (BiliNorm) to improve care for newborns with hyperbilirubinemia in Indonesia
Source: PLoS One. 2022 Jun 16;17(6):e0269286. doi: 10.1371/journal.pone.0269286 (PMC9202860; doi:10.1371/journal.pone.0269286)
Supplement: S1 Data — (PDF) [file pone.0269286.s001.pdf]

S1 Data. BiliNorm Questionnaire

Please fill out and complete the questionnaire according to the information listed below:

1 = Strongly disagree **5 = Slightly Agree**

2 = Disagree **6 = Agree**

3 = Slightly Disagree **7 = Strongly Agree**

4 = Neutral

	**Point**	**1**	**2**	**3**	**4**	**5**	**6**	**7**
**Utility**								
1.	BiliNorm helps you to determine phototherapy in jaundiced babies							
2.	BiliNorm helps you to pay more attention and be aware of Acute Bilirubin Encephalopathy							
3.	BiliNorm helps you to pay more attention and be aware of Kern Icterus							
4.	BiliNorm helps you for better management of hyperbilirubinemia							
5.	BiliNorm helps you provide better communication, information and education for parents and patients' families							
6.	BiliNorm helps you to do better patient follow-up							
**Ease of use**								
1.	Learning how to use BiliNorm is easy							
2.	BiliNorm easily understands what you want for better management of hyperbilirubinemia							
3.	BiliNorm is a clear and easy to understand							
4.	BiliNorm is flexible							
5.	It is very easy for you to become proficient in using BiliNorm							

6.	BiliNorm is an easy-to-use application							
**Subjective Norms**								
1.	Your supervisor and other colleagues will think that BiliNorm is an important application							
2.	The use of BiliNorm is important for your supervisor and other colleagues							
3.	If you stop using BiliNorm, this will not be a problem for your supervisor and other physicians							
4.	Your supervisor and other colleagues hope that you will use BiliNorm in the future							
5.	Your supervisor and other colleagues will be surprised if you stop using BiliNorm							
6.	Your supervisor and other colleagues will be disappointed if you stop using BiliNorm							
7.	Your supervisor and other medical colleagues will make you feel guilty if you stop using BiliNorm							
**Tendency to use BiliNorm**								
1.	You are likely to use BiliNorm in the next few months							
2.	You predict that you will continue to use BiliNorm in the next few months							
3.	You plan to use BiliNorm in the next few months							
