# Supplementary material for: Evaluation of a mobile application tool (BiliNorm) to improve care for newborns with hyperbilirubinemia in Indonesia
Source: PLoS One. 2022 Jun 16;17(6):e0269286. doi: 10.1371/journal.pone.0269286 (PMC9202860; doi:10.1371/journal.pone.0269286)
Supplement: Attachment — Submitted filename: Respond to Editor Revision.docx [file pone.0269286.s002.docx]

IMPORTANT: PLEASE DO NOT REPLY TO THIS EMAIL

If you are unable to complete any points that are requested in this email, please explain why in the 'Enter Comments' tab of the online submission form prior to re-submitting your manuscript. This will let us quickly assess your response and send your manuscript to an Academic Editor as soon as possible.

=============================================

PONE-D-21-37055R1

Evaluation of a mobile application tool (BiliNorm) to improve care for newborns with hyperbilirubinemia in Indonesia

Dr. Mahendra Tri Arif Sampurna

Dear Dr. Sampurna,

We've checked your submission and before we can proceed, we need you to address the following issues:

1. Thank you for stating the following financial disclosure:

“This project was supported by a research grant from the National Institute of Health Research and Development (NIHRD), Ministry of Health, Republic of In-donesia, Jakarta, Indonesia HK.03.01/I/1186/2019 which was granted to MTAS. The National Institute of Health Research and Development was responsible to provide financial assistance, as well as technical assistance through periodic review to ensure the research achieves the desired output.” Please state what role the funders took in the study.  If the funders had no role, please state: "The funders had no role in study design, data collection and analysis, decision to publish, or preparation of the manuscript." If this statement is not correct you must amend it as needed. Please include this amended Role of Funder statement in your cover letter; we will change the online submission form on your behalf.

**Respond : Thank you for your reminder, the statement is correct and we have added the statement on the cover letter.**

2. PLOS requires an ORCID iD for the corresponding author in Editorial Manager on papers submitted after December 6th, 2016. Please ensure that you have an ORCID iD and that it is validated in Editorial Manager. To do this, go to ‘Update my Information’ (in the upper left-hand corner of the main menu), and click on the Fetch/Validate link next to the ORCID field. This will take you to the ORCID site and allow you to create a new iD or authenticate a pre-existing iD in Editorial Manager. Please see the following video for instructions on linking an ORCID iD to your Editorial Manager account: <https://www.youtube.com/watch?v=_xcclfuvtxQ>

**Respond : Thank you for the information, now we already added our orcid id in our editorial manager.**

3. Please upload a copy of Figure 4, to which you refer in your text. If the figure is no longer to be included as part of the submission please remove all reference to it within the text.

**Respond : For the figure 4, we have added this figure in the Figures and Algorithm.docx, at the submission. Please kindly check it and remind me if it still does not appear in the submission. Thank you**

4. Please note that funding information should not appear in any section or other areas of your manuscript. We will only publish funding information present in the Funding Statement section of the online submission form. Please remove any funding-related text from the manuscript.

**Respond : Thank you for your information, we already remove any funding-related text from the manuscript.**

5. We note that your Data Availability Statement reads: "

“Authors are not able to make the underlying data set publicly available for legal or ethical reasons (based on, number, about). Data cannot also be shared publicly because of requiring agreement from multiple institutions. Possibility to obtain the data can be arranged to the interested party, however they should first contact the Ethical Committe of Dr Soetomo General Hospital for the initiating the data transfer and legality process."

We note that you have indicated that data from this study are available upon request. PLOS only allows data to be available upon request if there are legal or ethical restrictions on sharing data publicly. For information on unacceptable data access restrictions, please see http://journals.plos.org/plosone/s/data-availability#loc-unacceptable-data-access-restrictions.

Before we proceed with your manuscript, please address the following prompts:

A. Please provide the institutional body responsible for imposing the restrictions upon your data (e.g., a Research Ethics Committee or Institutional Review Board, etc.)

B. Please note that it is not acceptable for authors of the study to be either the sole listed point of contact for data or the ultimate provider of data that is made available upon request. At this time, please provide direct, non-author contact information (preferably email) for the body imposing the restrictions upon the data, to which data access requests can be sent.

We will update your Data Availability statement upon your response.

**Respond : Thank you for your reminder. We understand that it is important to have the data set available. However, we are not able to make the underlying data set publicly available for legal or ethical reasons. Data cannot also be shared publicly because of requiring agreement from multiple institutions. We believe that our reason to not share the data doesn't fall within the criteria of unacceptable data restriction. Our data is quite sensitive because we discuss undertreatment and overtreatment that risk the reputation of an institution. Here we provide the number of the Ethical Committee of Dr Soetomo General Hospital (+62 82143729965), for further information.**

We've returned your manuscript to your account. Please resolve these issues and resubmit your manuscript within 21 days. If you need more time, please email the journal office at plosone@plos.org. We are happy to grant extensions of up to one month past this due date. If we do not hear from you within 21 days, we will withdraw your manuscript.

Please log on to PLOS Editorial Manager at https://www.editorialmanager.com/pone/ to access your manuscript. You will find your manuscript in the 'Submissions Sent Back to Author' link under the New Submissions menu. Be sure to remove your previous manuscript file if you are uploading a new file in response to these requests. After you've made the changes requested above, please be sure to view and approve the revised PDF after rebuilding the PDF to complete the resubmission process.

We are requesting these changes to comply with the PLOS ONE submission guidelines (https://journals.plos.org/plosone/s/submission-guidelines). Please note that we won't send your manuscript for review until you have resolved the above requests.

Thank you for submitting your work to PLOS ONE and supporting our mission of Open Science.

Kind regards, Rose Ann Joyce Sagun Puetes PLOS ONE
